# Discrete partitioning of HIV-1 Env forms revealed by viral capture

**DOI:** 10.1186/s12977-015-0207-z

**Published:** 2015-09-24

**Authors:** Daniel J. Stieh, Deborah F. King, Katja Klein, Yoann Aldon, Paul F. McKay, Robin J. Shattock

**Affiliations:** Department of Cellular and Molecular Biology, Feinberg School of Medicine, Northwestern University, Chicago, IL 60611 USA; Mucosal Infection and Immunity Group, Section of Infectious Diseases, Imperial College London, St Mary’s Campus, London, W2 1PG UK

**Keywords:** HIV-1, Monoclonal antibody, Envelope glycoprotein, Viral heterogeneity

## Abstract

**Background:**

The structure of HIV-1 envelope glycoprotein (Env) is flexible and heterogeneous on whole virions. Although functional Env complexes are thought to require trimerization of cleaved gp41/gp120 heterodimers, variable processing can result in the potential incorporation of non-functional uncleaved proteins (gp160), non-trimeric arrangements of gp41/gp120 heterodimers, and gp120 depleted gp41 stumps. The potential distribution of functional and non-functional Env forms across replication-competent viral populations may have important implications for neutralizing and non-neutralizing antibody functions. This study applied an immuno-bead viral capture assay (VCA) to interrogate the potential distribution (heterologous vs homologous) of functional and non-functional forms of virion associated Env.

**Results:**

The VCA revealed a significant association between depletion of infectious virions and virion Env incorporation, but not between infectivity and p24-gag. Three distinct subpopulations of virions were identified within pools of genetically homogenous viral particles. Critically, a significant subpopulation of infectious virions were exclusively captured by neutralizing antibodies (nAbs) indicative of a homologous distribution of functional trimeric Env forms. A second infectious subpopulation bound both neutralizing and non-neutralizing antibodies (nnAbs) representative of a heterologous distribution of Env forms, while a third non-infectious subpopulation was predominantly bound by nnAbs recognizing gp41 stumps.

**Conclusions:**

The observation that a distinct and significant subpopulation of infectious virions is exclusively captured by neutralizing antibodies has important implications for understanding antibody binding and neutralization, as well as other antibody effector functions.

**Electronic supplementary material:**

The online version of this article (doi:10.1186/s12977-015-0207-z) contains supplementary material, which is available to authorized users.

## Background

Successful vaccination to prevent HIV-1 acquisition will likely require the elicitation of neutralizing antibodies (nAb) directed against the functional envelope glycoprotein (Env) [1–3]. However, although natural infection rapidly induces Env specific class switched B cells [4], these lack the required specificity to provide potent and broad viral neutralization. Indeed maturation of autologous nAbs requires months to years of persistent systemic infection [3], where <1 % of individuals develop substantial breadth of heterologous neutralization (elite neutralizers) [5–7]. Thus the features of Env variants that lead to neutralization breadth remain to be defined.

The predominance of antibodies that fail to neutralize HIV-1 in infected individuals is thought to be due in part to the diversity of Env conformations presented to the humoral immune system. Virion Env diversity is generated by multiple mechanisms including: the range of virion incorporated Env structures, the degree and extent of Env glycosylation, the flexible nature of the HIV-1 Env protein, and potential instability on the surface of viral particles [8–10].

HIV-1 Env is expressed in infected cells as a non-functional precursor protein gp160. Subsequent proteolytic cleavage of gp160 by cellular furins into non-covalently attached transmembrane gp41 and external gp120 components is a necessary step in the creation of functional Env [11]. The non-covalent nature of the cleaved gp41/gp120 protein provides an intrinsic instability that is distinct from heterodimeric Env proteins of other viruses such as influenza, which retains a stabilizing disulfide bond [12]. The instability of the gp41/gp120 heterodimer can lead to gp120 shedding leaving gp41 stumps expressed on the virus or infected cells. Although functional Env complexes are thought to require gp41/gp120 trimerization, variable gp160 processing results in the expression of uncleaved proteins and non-trimeric arrangements of gp41/gp120 heterodimers on the surface of infected cells [13]. Assembly of these Env structures into budding virions provides further variability, with gp41/gp120 incorporated as monomeric, dimeric and trimeric forms [13–15]. Additional diversity is derived from the range of gp120 glycosylation patterns that can obfuscate the Env spike from humoral responses. The array of gp120 N-linked oligomannose glycans is inefficiently trimmed by Golgi and Endoplasmic Reticulum α-mannosidases [16, 17], and this is further reduced by the steric constraints imposed by trimerization [18].

The plasticity of the Env complex itself leads to variable and transient epitope exposure further confounding the potential elicitation of nAb responses [19]. This is enhanced by the fact that a series of conformational rearrangements occur during receptor triggering and viral-cell fusion [20, 21]: the co-receptor (CCR5 or CXCR4) binding site is exposed after binding to the CD4 molecule; and the fusion peptide of gp41, that inserts into the target cell and initiates membrane fusion, is only fully exposed following CD4 and co-receptor binding.

The inherent flexibility and high potential energy of the Env complex is likely necessary for correct functioning, but this complicates the development of effective mimic immunogens for use in vaccination regimes. Nonetheless, a growing number of broadly neutralizing monoclonal antibodies (mAbs) have been isolated from a few elite controllers that map to sub-regions on the Env spike [22–24]. These include PG9, PG16, VRC01 and VRC03 mAbs that are understood to recognize gp120 in the context of functional trimers, and display very broad breadth of neutralization in comparison to the previously identified CD4 binding site (CD4bs) b12 mAb and glycan specific 2G12 mAb [25, 26]. Additional very potent broadly nAbs (bnAbs) targeting gp120 have recently been described [22, 27]. In rare instances nAbs have also been isolated from infected subjects that map to the gp41 membrane proximal external region (MPER), including 4E10, 2F5 and 10e8 mAbs [28–31].

Although binding to functional oligomeric Env is thought to be essential for neutralization, the extent to which bnAb discriminate between functional and non-functional Env is not fully defined. Furthermore, the distribution of variant Env forms across replication competent viral populations may have profound implications for nAb and nnAb function [32]. It remains unclear whether functional and non-functional Env variants are distributed randomly or whether there is differential partitioning forming discrete subpopulations of virions. Indeed, the extent to which individual virions are heterologous or homologous with respect to expression of functional and non-functional Env is unknown. This has functional significance as only a fraction of these structures may present epitopes that are susceptible to antibody neutralization, while non-functional forms may be subject to binding by nnAbs and subsequent virion opsonization rendering virions susceptible to FcR effector functions including antibody-dependent cellular phagocytosis (ADCP) [33]. In this study, using an immuno-bead based viral capture assay (VCA) and a panel of antibodies of known specificities, we investigate the potential distribution (heterologous vs homologous) of functional and non-functional forms of virion associated Env.

## Results

### Env-specific mAbs capture varying levels of total viral particles and gp120 positive virions

An immuno-bead VCA was used to interrogate variability in the structure and conformation of Env glycoprotein incorporated into intact virions. Viral capture was assessed using a panel of nAb and nnAb, as well as mAbs to host derived proteins HLA-DR and CD45, which have been reported as selectively included or excluded from viral particles respectively. mAbs were incubated with highly purified viral preparations, followed by incubation with protein G coupled paramagnetic beads to capture antibody-coated virus. The antibody-bead labeled positive fraction was then separated from free virions (negative fraction) by retention on a magnetic column. Specific protein content (p24 or gp120) of the retained positive fraction was expressed as a percentage of the sum of the positive and negative fractions combined. Virion-associated p24, considered as evenly distributed amongst virions, was used to quantify the total amount of virus present. The use of purified viral preparations, free from non-viral associated p24, enabled quantitation of virions in the labeled fraction as a portion of the whole. Virion-associated gp120 was used to quantify capture of gp120 positive virions.

**Fig. 1:**
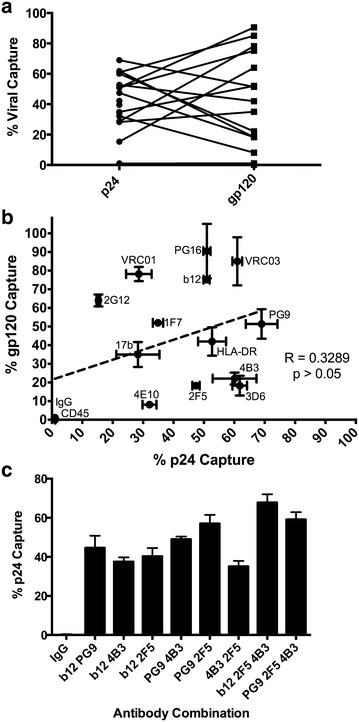
Capture of purified virus by a panel of monoclonal antibodies against Env epitopes and host-derived proteins. Purified HIV-1_BaL_ virions incubated with a range of antibodies were then retained on magnetic protein G microbeads. The retained, positively bound fraction was expressed as a proportion of the total protein, quantified by either p24 or Env capture ELISA. **a** Pair-wise representation of the antibody binding to virions when quantified by p24 or gp120 capture. The Wilcoxon matched pairs T Test between p24 and gp120 was insignificant, p = 0.6495. **b** There was no significant correlation between increased % p24 capture with % gp120 capture. **c** Combined antibodies in capture assays were quantified by p24 retention, and show an additive effect of combining antibodies targeting multiple Env epitopes. Capture assays were performed in triplicate, and repeated three times. *Error bars* represent the standard deviation

Both total virion capture (p24) and capture of gp120 positive virions varied by antibody (Table 1). There was no correlation between whole virion (p24) and gp120 capture (Fig. 1a) and no predictive value of relative capture from one parameter to the other (Spearman R = 0.3289, p > 0.05, Fig. 1b). This is unsurprising given that virions homologous for gp41 stumps would not be captured by antibodies to gp120. Indeed antibodies targeting gp41 epitopes in MPER and cluster I (C1) were associated with a low gp120:p24 ratio (<1), while those targeting exposed epitopes on trimeric Env were generally associated with ratio’s >1. These data suggest differential depletion of a mixed population of virions expressing different Env forms where no individual antibody could capture all virions.

**Table 1:** Viral capture of HIV-1 with a panel of monoclonal antibodies measured by p24 or gp120

Antibody	Binding epitope	% p24	% gp120	gp120:p24
b12	gp120 CD4bs	50.9 (1.0)	75.2 (1.3)	1.48
b6	gp120 CD4bs	34.2 (7.9)	23.8 (3.7)	0.70
1F7	gp120 CD4bs	34.9 (1.7)	52.0 (1.8)	1.49
VRC01	gp120 CD4bs	28.6 (4.2)	78.2 (3.8)	2.73
VRC03	gp120 CD4bs	61.0 (1.6)	85 (12.9)	1.39
17b	gp120 CD4i	28.3 (7.1)	35.0 (6.7)	1.24
PG9	gp120 V2/V3 loop	69.0 (5.2)	51.4 (7.9)	0.74
PG16	gp120 V2/V3 loop	51.0 (1.2)	90.5 (14.6)	1.77
2G12	gp120 mannose	15.3 (0.6)	64.0 (3.2)	4.18
4B3	gp41 cluster I	60.1 (7.2)	22.1 (3.2)	0.37
3D6	gp41 cluster I	61.8 (2.4)	18.3 (5.3)	0.30
F240	gp41 cluster I	52.7 (8.1)	ND	ND
7B2	gp41 cluster I	39.6 (2.4)	ND	ND
5F3	gp41 cluster V	42.2 (9.4)	ND	ND
2F5	gp41 MPER	47.4 (1.2)	18.3 (1.3)	0.39
4E10	gp41 MPER	32.1 (2.3)	8.1 (0.5)	0.25
HLA-DR	Host protein	52.7 (4.6)	42.0 (7.6)	0.80
CD45	Host protein	1.1 (0.1)	0.1 (0.1)	0.09
IgG isotype	Non-specific	1.0 (0.0)	1.1 (0.1)	1.10

Viral capture measured by particle and Env retention. Data shown are means and standard deviation, with n ≥ 3

*ND* not determined

**Fig. 2:**
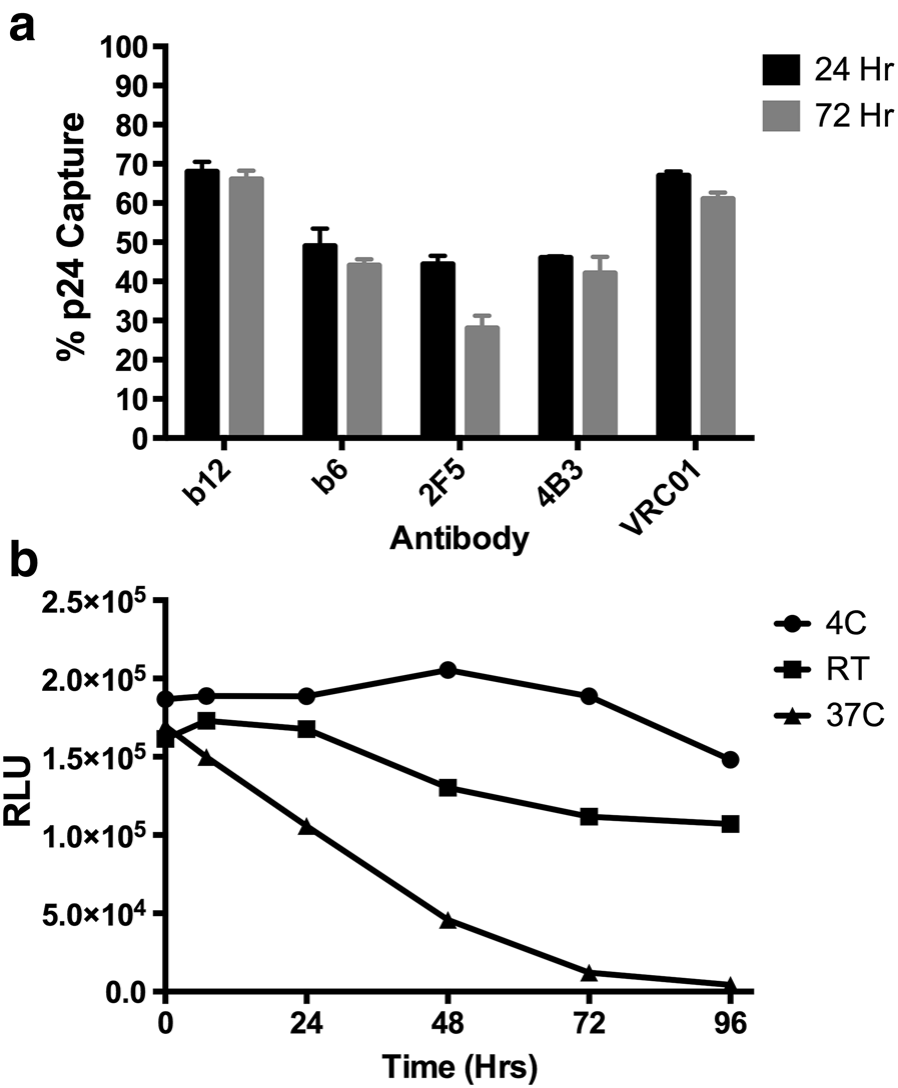
Changes in viral integrity determined after **a** timing of virus harvest or **b** incubation at a range of temperatures. **a** Viral capture, as described in Fig. 1 legend, with 5 antibodies was determined by % p24 capture when virus was harvested 24 or 72 h after final media change. **b** Viral infectivity was measured on TZM-bl cells after virus was stored at the indicated temperature for up to 96 h. Data shown is the average and standard deviation of two experiments, each performed in triplicate

**Fig. 3:**
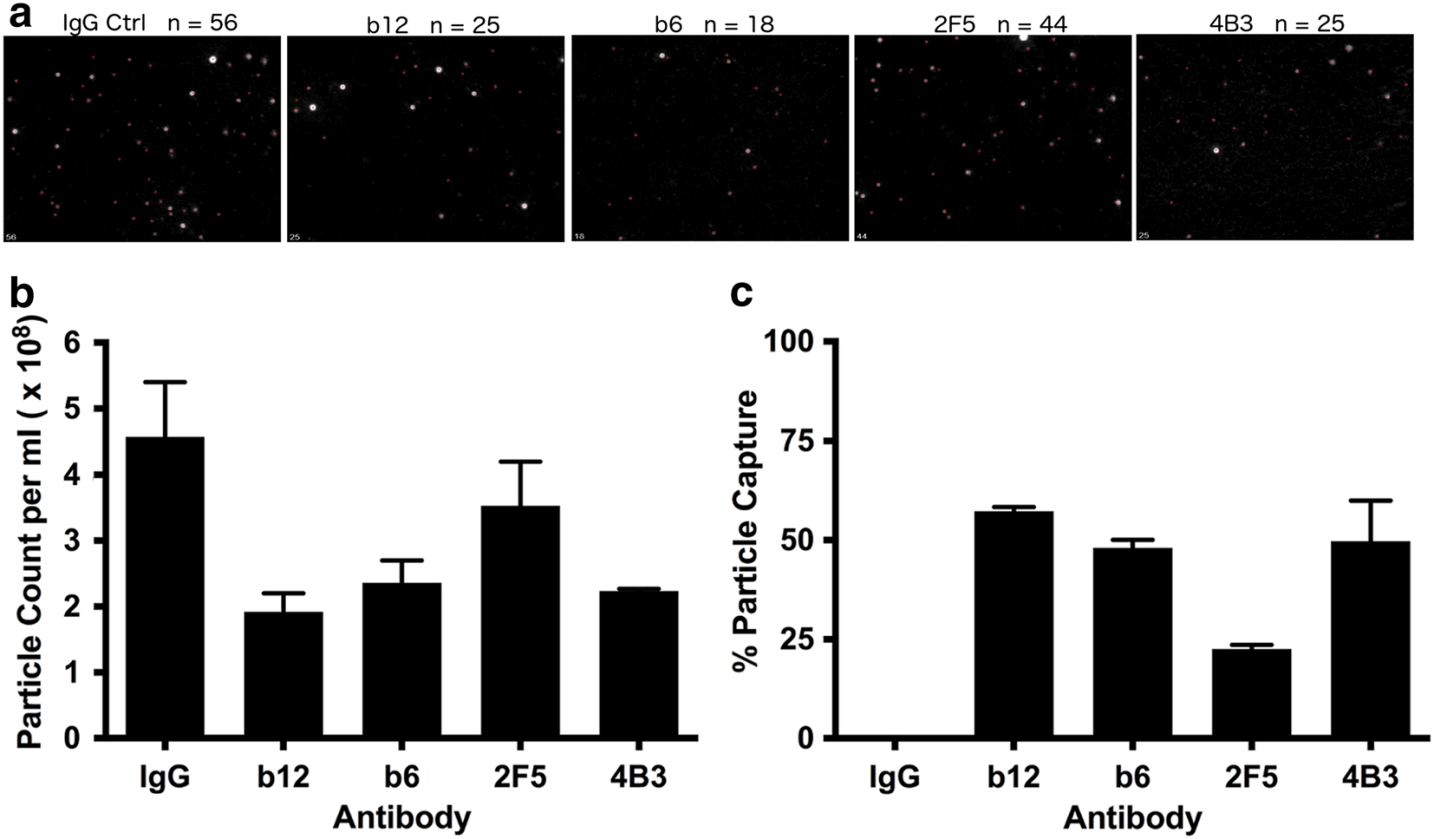
Direct visualization and quantitation of viral particles after antibody depletion. **a** Nanoparticle tracking analysis (Nanosight) of HIV-1_BaL_ was performed after depletion by 5 monoclonal antibodies. Representative still images from movies are shown. The number of virions identified by *red makers* is indicated. Nanosight quantification of (**b**) total particle counts and (**c**) % capture of viral particles after sorting. Data shown is the average of at least two independent experiments

There was negligible decrease in total virion capture (p24) between virus harvested after 24 or 72 h of culture in fresh media (p = 0.068, ANOVA), indicating that the differences in the relative proportions of virus capture between antibodies (gp120:p24) were an inherent aspect of early viral particle production and did not arise from a progressive degradation of Env in culture (Fig. 2a). Degradation of virus was not seen to occur during timescales of VCA, requiring sustained incubation at elevated temperature to degrade infectivity (Fig. 2b). Combining mAbs that recognize gp120 (b12 or PG9) and gp41 (4B3 and 2F5) together resulted in enhanced capture with the majority of virions being bound (Fig. 1c), confirming that measured p24 was exclusively derived from intact viral particles. The efficiency of viral particle capture was also confirmed for select antibodies (b12, b6, 2F5, and 4B3) by nanoparticle tracking analysis that allows direct physical quantification of virion particles (Fig. 3) [34]. These results correlated significantly with the quantification of total viral particles assessed by p24 content (Spearman R = 0.9000, p < 0.05).

### Capture of virions with varying Env conformation and cleavage states is determined by antibody specificity

**Fig. 4:**
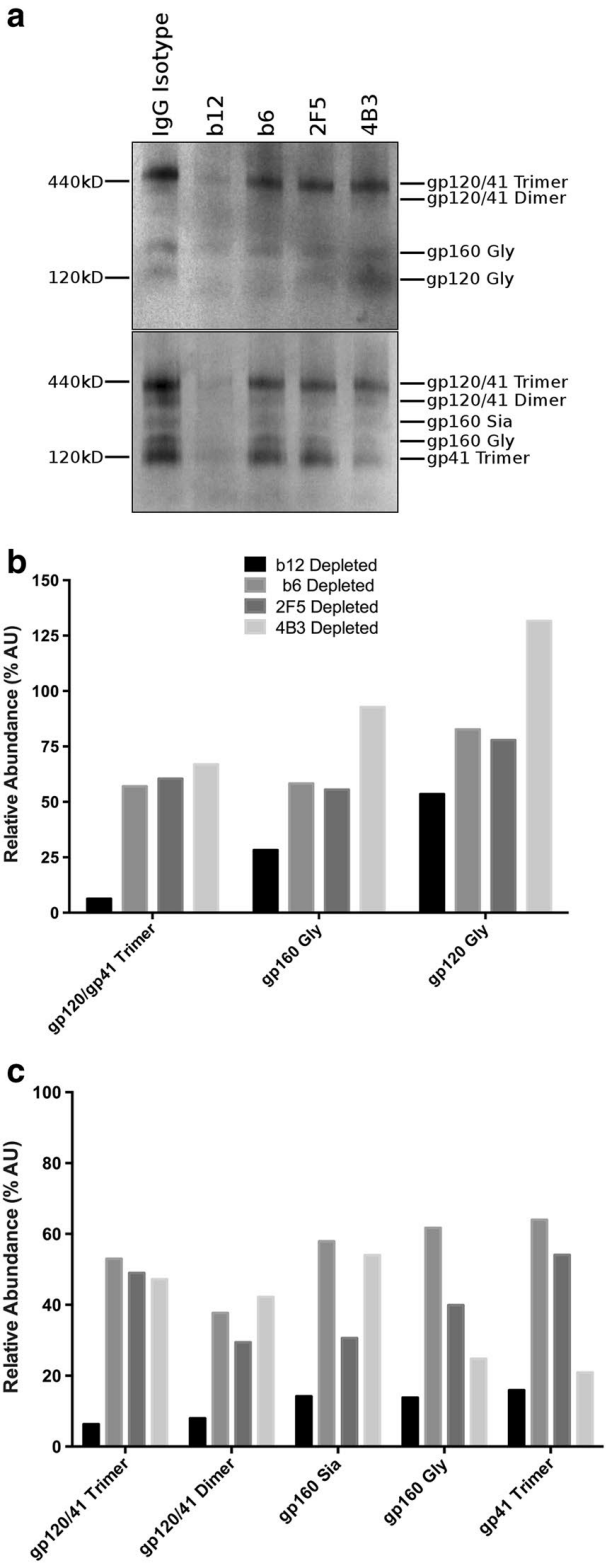
Native-PAGE determination of the Env conformations present after virus depletion. **a** Purified HIV-1_BaL_ virions were depleted by the antibody specified and separated on native-PAGE gels and western blots were probed with a gp41 (2F5, 4B3, 3D6) or gp120 (b12, 2G12, b6) antibody cocktail. The abundance of Env species were quantified relative to undepleted virus for blots probed with (**b**) gp120 antibodies or (**c**) gp41 antibodies. Blot is representative of at least 3 independent depletion experiments and quantification is the average of 3 images

Native PAGE was used to further understand the differential depletion of mixed populations of virions by select antibodies. Trimeric gp41/gp120 was the most abundant form of Env on purified virions (Fig. 4a), with lower levels of dimeric and monomeric forms as well as uncleaved gp160. Viral capture with b12, in concord with its preferential recognition of the CD4bs on trimeric Env, preferentially depleted gp41/120 trimers from the viral stock, and also had the largest impact on total Env depletion (Fig. 4b, c). In contrast, b6 depletion, which binds with lower affinity to the CD4bs on trimeric Env [35], was inefficient at depleting trimeric gp41/120 relative to other forms of Env. MPER specific 2F5 was similarly inefficient at depleting trimeric gp41/120 relative to other forms of Env. Furthermore, while capture using gp41 (cluster I) specific 4B3 efficiently depleted gp41 trimers devoid of gp120 (Fig. 4c), it was the least efficient at depleting structures containing gp120 or gp160 (Fig. 4b). These data are in agreement with the relative proportions of total gp120 detected by ELISA (Table 1).

**Fig. 5:**
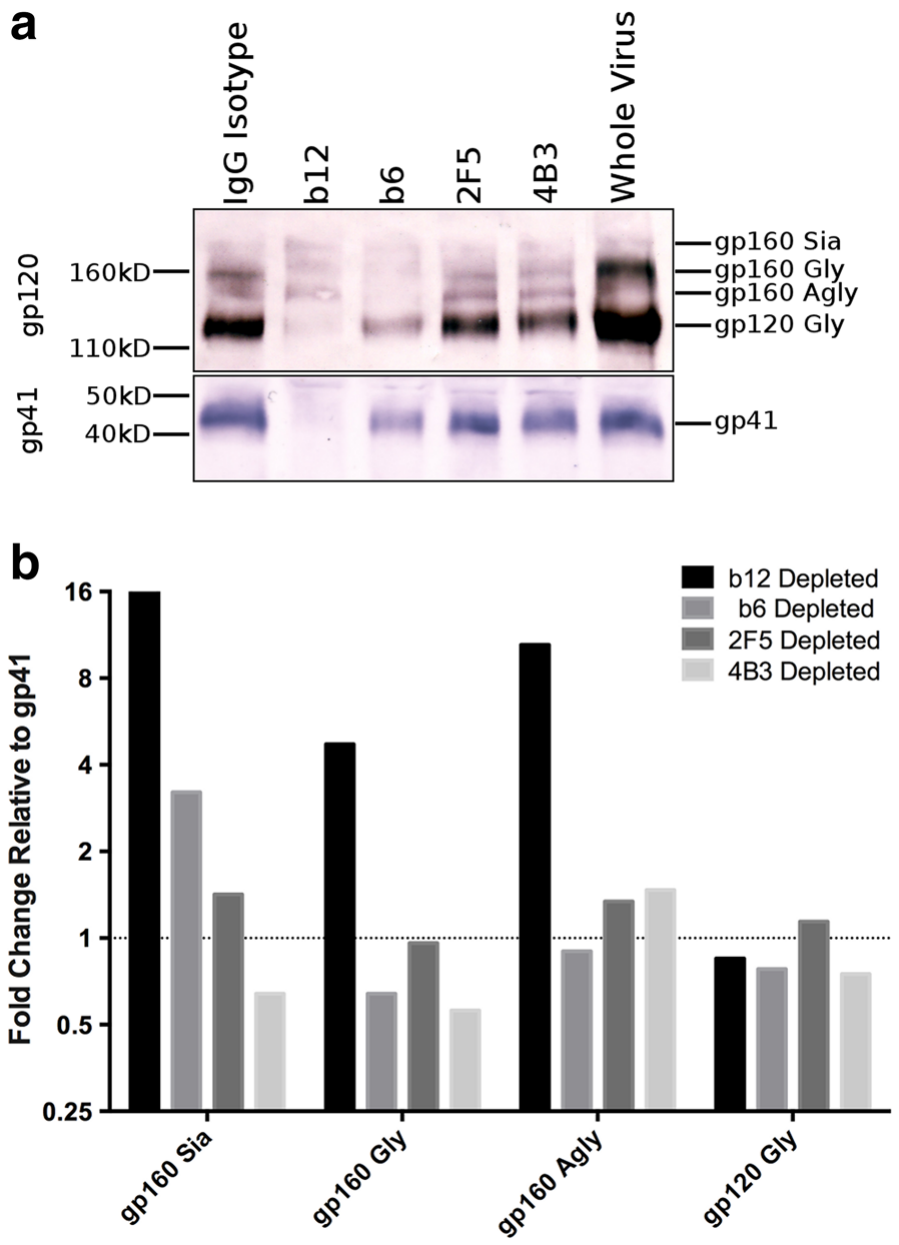
SDS-PAGE determination of the relative binding to Env states after virus depletion. **a** Purified HIV-1_BaL_ virions were depleted and separated on SDS-PAGE gels and western blots were probed with a gp41 (2F5, 4B3, 3D6) or gp120 (b12, 2G12, b6) antibody cocktail. **b** The ratio of each species identified in the gp120 probed blot relative to gp41 is used to determine preferential binding to Env states. Blot is representative of 3–5 independent experiments and quantification is the average of 3 images

Parallel analysis was performed by SDS-PAGE (Fig. 5a). Cleaved gp120 (gp120 Gly) was the predominant species detected by gp120 mAbs on untreated purified virions with a lower proportion of uncleaved gp160 forms (Fig. 5a). These data concord with native PAGE where the predominant form of virion-associated Env was trimeric gp41/120. Viral capture with b12 resulted in over 90 % depletion of gp120 Gly and gp41, confirming the preferential depletion of trimeric gp41/120 by native PAGE. b12 capture resulted in lower depletion of gp160 Gly, and only minor depletion of alternatively glycosylated gp160 forms. Comparison of fold change in gp120/gp160 species relative to gp41 revealed an enrichment of all gp160 species following b12 capture (Fig. 5b). This indicates preferential depletion of a distinct subpopulation of viral particles within the viral stock: those expressing cleaved gp41/120—captured by b12, and those expressing uncleaved gp160 that as a consequence of preferential gp41/120 depletion were enriched relative to gp41. By contrast, b6 and 4B3 capture displayed a modest preferential targeting of uncleaved gp160 resulting in a small reduction in gp160 forms relative to gp41. 2F5 capture had no effect on the ratio of gp120/160:gp41 reflecting a modest reduction in all Env forms.

### Env-specific mAbs bind to and capture varying amounts of infectious HIV-1

**Fig. 6:**
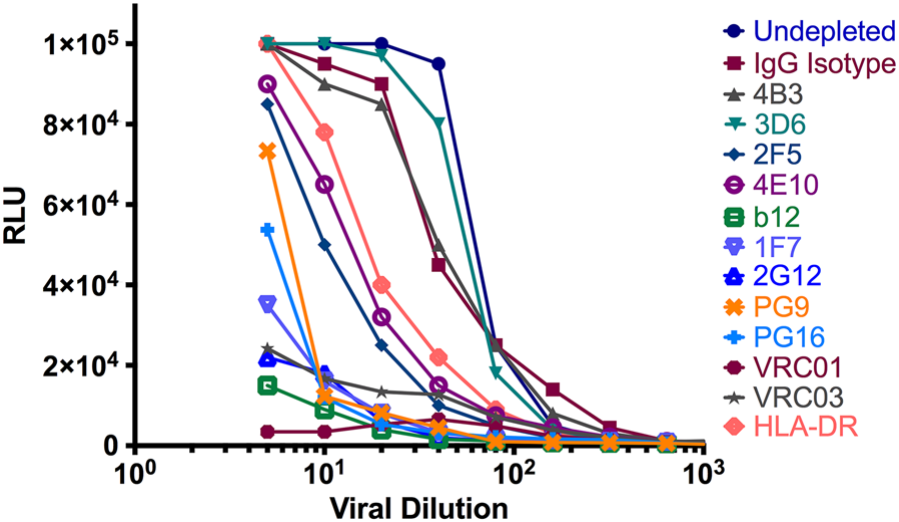
Depletion of infectious particles with a panel of Env-specific, or host protein directed mAbs. Infectious stock of HIV-1_BaL_ was depleted with a range of antibodies. The remaining depleted virus was titrated on TZM-bl cells and compared to virus treated with isotype controls. The area under the curve relative to the IgG control depleted preparation is used to determine % infectivity depletion. Data represent the mean of three independent experiments each performed in triplicate

The VCA was then modified to assess depletion of infectious HIV-1_BaL_ virions. Here, virus was incubated with antibody of interest, followed by depletion with protein G beads. The bound fraction was retained on a column and the eluted virus assessed for infectivity. Depletion of infectious virus was determined as percentage change in the area under the curve relative to the IgG isotype control. In line with their specificity for functional forms of Env, neutralizing antibodies targeting gp120 captured and consequently efficiently depleted infectious particles (Fig. 6). In contrast antibodies preferentially targeting gp41 or non-trimeric forms of Env (b6) were ineffective (Fig. 6, Additional file 1: Figure S1).

**Fig. 7:**
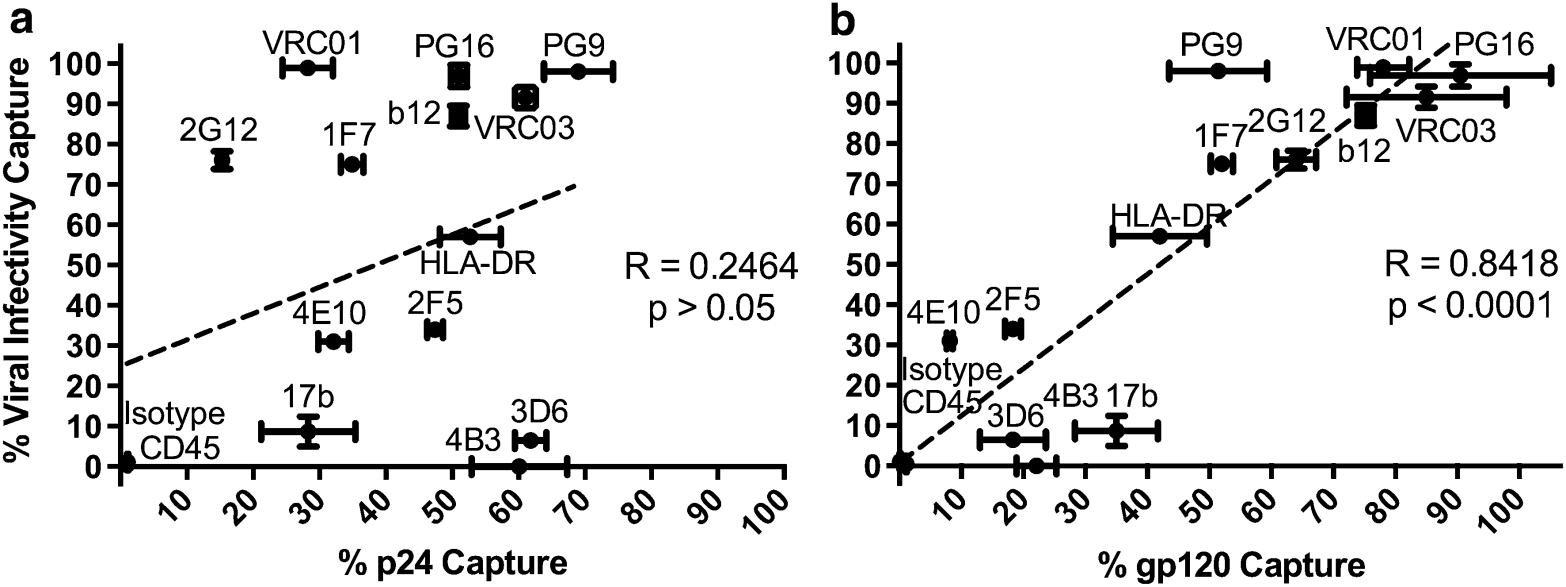
Correlation between **a** % p24 or **b** % gp120 capture and % infectivity capture by a panel of Env-specific mAbs are shown. Spearman correlation between the amount of % p24 captured and depletion of infectivity was insignificant (Spearman R = 0.2464, p > 0.05) but highly significant correlation exists between % gp120 binding and % infectivity capture (Spearman R = 0.8418, p < 0.01)

For HIV-1_BaL_ there was no correlation between p24 and infectivity capture (R = 0.2464, p > 0.05; Fig. 7a), similar to the lack of association between p24 and gp120 positive populations. However, a strong correlation was found between the gp120 positive population and infectivity depletion (Spearman R = 0.8418, p < 0.0001; Fig. 7b). These data are concordant with trimeric Env being the predominant form within the virion population. The ability of antibodies VRC01, 2G12 and IF7 to capture over 90 % of infectious virions but less than 35 % of viral particles (p24) demonstrates that functional Env molecules are not evenly distributed amongst virions. Furthermore, the inability for antibodies preferentially targeting non-functional forms of Env to capture significant amounts of infectious virus, while capturing a significant proportion of total virions (p24) provides further evidence for distinct viral subpopulations: those expressing functional Env and consequently infectious, and non-infectious virions expressing non-functional forms. Subsequent experiments were designed to determine the extent of potential overlap in the distribution of these different Env forms on virions within the same viral population.

Because HIV-1_BaL_ is a lab-adapted virus we next considered depletion of primary strains. Similar levels of infectious capture were seen for a smaller panel of nAbs tested against clade B primary clinical isolate Bx08 (Additional file 2: Figure S2A). With the clade C transmitted/founder virus CH162.c, there was a weak positive correlation between retaining virus particles (p24) and infectious virions (Spearman R = 0.5711, p = 0.04; Additional file 2: Figure S2B). Capture of virus particles (p24) and gp120 were not significantly correlated (Spearman R = 0.4020, p = 0.06; Additional file 2: Figure 2C). A highly significant positive correlation existed between capture of infectious particles and g120 (Spearman R = 0.6544, p < 0.01; Additional file 2: Figure 2D). While strain specific differences exist in epitope exposure, capture of infectious particles revealed a similar pattern of depletion as HIV-1_BaL_ by bnAbs PG9, PG16, PGT151 and VRC01 that depleted more than 80 % of infectious virions. Other mAbs were capable of capturing substantial amounts of viral particles but not preferentially binding to infectious virions or gp120 (7B2, 4B3, 4E10, 5F3). However, mAbs with a high degree of p24 capture did not necessarily retain much infectious virus and the strongest association was between capture of gp120 and infectious virions. No antibody activity was present in the depleted fraction ruling out potential neutralization of the eluted fraction (Additional file 3: Figure S3).

### Sequential sorting of virions reveals three overlapping states of Env on viral particles

Two sequential rounds of viral capture were used to determine the potential overlap of functional and non-functional Env forms within the viral population. First round viral capture was used to deplete virions expressing accessible gp41 C1 forms of Env (4B3 capture), functional trimers (b12 capture) or exposed MPER epitopes (2F5 capture), using a standardized viral input of 1000 ng p24. 4B3 bound 60 % (601 ng) of virus (p24), 2F5 bound 47 % (474 ng), while b12 bound 51 % (509 ng) (Table 2, first round capture). The residual depleted populations were collected and subject to a second round of antibody capture and the bound fractions determined (Table 2). When the same antibody was used in first and second round capture minimal virus was bound confirming that the first round of depletion was >99 % effective.

**Table 2:** Sequential sorting of viruses with antibodies against varying HIV-1 Env epitopes, measured by p24 capture in ng/p24 and as a percentage of p24 input

Capture mAb	First round capture		4B3 Depleted Second round capture		b12 Depleted Second round capture		2F5 Depleted Second round capture	
	Input: 1000 ng		Input: 399 ng		Input: 491 ng		Input: 526 ng	
	ng/p24	% input	ng/p24	% input	ng/p24	% input	ng/p24	% input
4B3	*601*	*60.1*	0.8	0.2	457.6	93.2	373.5	71.0
2F5	*474*	*47.4*	92.2	23.1	206.2	42.0	4.2	0.8
b12	*509*	*50.9*	375.9	94.2	1.0	0.2	260.4	49.5

The header row describes the input virus, either total virus for the first round capture (1000 ng/p24) or the collected unbound fraction following first round depletion with 4B3 (399 ng/p24), b12 (491 ng/p24) or 2F5 (526 ng/p24)

Italics indicate the values after first round capture

**Fig. 8:**
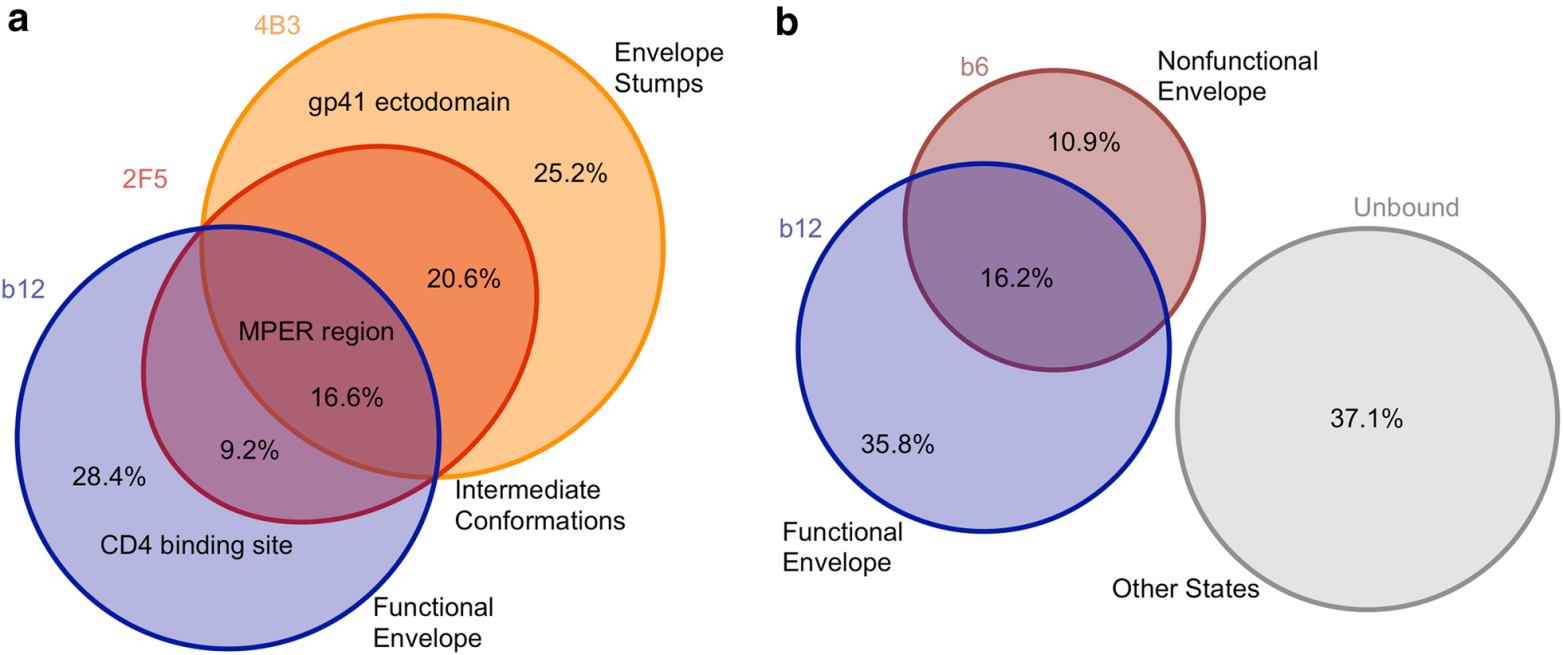
Venn diagram of the overlapping states of Env present on viral particles. Sequential viral sorting of purified HIV-1_BaL_ virions as described in Fig. 1 legend, with either **a** 4B3, 2F5 and b12 or **b** b12 and b6 was quantified by p24 ELISA. The overlap between populations was determined by the change in % and total amount of p24 captured after a first round of depletion relative to the p24 capture of purified viral preparations. Data shown is the average of three experiments, performed in triplicate

Sequential capture with b12 and 4B3 (in either order) bound 98 % of all virions indicating that the total MPER accessible fraction (2F5) was represented within these two populations. Taking this into account it was possible to calculate the relative abundance of gp41 C1, CD4bs and MPER single, dual and triple positive virions within the initial viral population. Within the initial viral population 28.4 % of virions were CD4bs single positive (b12), 25.2 % expressed only gp41 C1 (4B3), 20.6 % were dual positive for gp41 C1 and MPER (2F5), 9.2 % double positive for MPER and CD4bs, and 16.6 % triple positive for gp41 C1, MPER and CD4bs (Fig. 8a). Thus >50 % of virions expressing functional Env (b12+) were negative for non-functional forms, 17 % were dual positive for CD4bs and MPER, and 30.6 % were triple positive for CB4bs, MPER and gp41 C1.

In a second experiment, sequential rounds of viral capture were performed with the CD4bs antibodies b12 and b6, preferentially recognizing functional and non-functional forms of gp120 respectively (Fig. 8b). First round b6 capture bound 27.1 % of virions, equivalent to 271 ng of p24-associated virus from an initial input of 1000 ng/p24. Secondary capture with b12 bound 49.1 % of the unbound b6 negative fraction (358 ng/729 ng). b12 used in first round capture bound 52 % of the original initial non-depleted stock. Taking this into account we calculated the relative abundance of b6 and b12 single and dual positive populations within the viral population. Overall, 16.2 % of virions expressed both b6 and b12 CD4bs epitopes, 10.9 % expressed only b6, 35.8 % expressed only b12, and 37.1 % expressed neither CD4bs epitopes. This is consistent with b12 and b6 binding to largely distinct forms of Env while both targeting the CD4bs, and indicates that >68 % of virions positive for functional Env (b12+) were negative for non-functional forms of gp120 (b6−), while 48 % of virions in the total population expressed non-functional forms (b6−/b12− and b6+/b12−).

### Depletion of non-infectious virions has minimal impact on viral neutralization

**Fig. 9:**
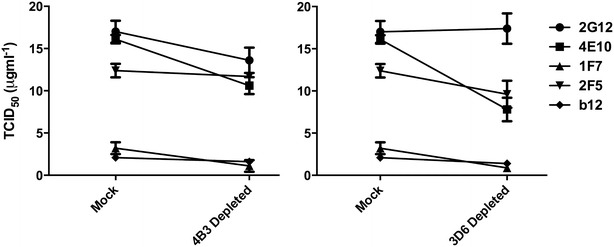
Effect on neutralization microvesicles was performesensitivity following depletion of non-infectious virions by 4B3 and 3D6. The IC_50_ of a panel of neutralizing antibodies against HIV-1_BaL_ was measured against mock depleted viral stocks and the same virus after having been depleted by 4B3 or 3D6. The depleted virus was tested by serial dilution of antibody on TZM-bl cells. Data shown is the average of three experiments, performed in triplicate. *Error bars* indicate standard deviation

Subsequent experiments were performed to assess the potential ‘decoy’ effect of non-functional Env on antibody neutralization. The relative inhibitory potency of a panel of nAbs (2F5, 4E10, b12, 1F7 and 2G12) was assessed following depletion with 3D6 or 4B3 that primarily recognize non-functional Env spikes or gp41 stumps (Fig. 9). Depletion with 3D6 or 4B3 had no significant impact on neutralization of HIV-1_BaL_ by gp120 specific nAbs: 2G12, b12 or 1F7, confirming lack of significant overlap between these populations. In contrast, the neutralizing potency of 4E10 was strongly affected after depletion with either 4B3 or 3D6 epitope expressing virions (p < 0.01, ANOVA, for both 4B3 and 3D6 depletion). This pattern was followed for 2F5 as well, with a statistically significant decrease in IC_50_ following 3D6 depletion (p < 0.01, ANOVA) from 12.4 to 9.6 μg ml^−1^ and a trend towards increased activity following 4B3 depletion (p = 0.063). Furthermore, when comparisons were made by ANOVA, where the 95 % confidence interval for the change in IC_50_ are calculated, following 4B3 depletion the only mAbs that resulted in confidence intervals excluding zero were 2F5 and 4E10. These data suggest that removal of virions expressing gp41 C1 epitopes can modestly enhance the neutralizing potency of MPER antibodies, confirming the significant overlap in expression of these two epitopes within the viral population, but had an insignificant impact on nAbs targeting gp120.

## Discussion

This study interrogates the distribution (heterologous vs homologous) of functional and non-functional forms of virion associated Env across viral particles. The use of an immuno-bead VCA enables quantitation of both bound and unbound fractions, which in turn allows quantitation of relative epitope exposure on intact virions. Although in-solution viral binding assays have been reported before [35, 36], this approach varies from previous plate based VCA in that all components of the system are in the native orientation that would be present during an in vivo binding event. By using depletion rather than a positive selection process, we are able to characterize subpopulations across replication-competent viral populations. Furthermore, the use of purified virions allows determination of virion associated gp120 and p24 protein in the antibody associated and unbound fractions after capture without confounding effects of free p24 or gp120. Multiple low-affinity, slow and/or transient binding events are minimized by incubation times sufficient for viral-antibody interactions to reach equilibrium (98 % within 1600 s for antibodies of a similar affinity at 10 μg ml^−1^, as calculated according to Hulme et al. [37]) in the liquid phase before capture by protein G magnetic beads.

The capture of p24 in this model did not correlate with the capture of infectious virions in agreement with previous observations [15], and confirms that a significant population of virions do not express functional Env [13, 38]. Indeed the ability of antibodies VRC01, 2G12 and IF7 to capture over 90 % of infectious virions but less than 35 % of viral particles (p24) demonstrates that functional Env molecules are not evenly distributed amongst virions (Table 1). Furthermore, no single antibody captured all virus, as assessed by p24 content or nanoparticle tracking analysis, suggesting no single epitope was expressed (or accessible) across all virion forms within the population. Interestingly, antibody to HLA-DR captured 52 % of virions suggesting that HLA incorporation is not as uniform or ubiquitous across all viral particles as perhaps previously thought [39, 40] (Table 1). In contrast there was a positive and highly significant correlation between gp120 and infectivity. nAbs targeting gp120 epitopes were highly efficient at capture of both infectious virus and gp120 containing virions. However, mAbs thought to require receptor triggered conformational change to expose their cognate epitopes on gp120 (17b) or gp41 (2F5 and 4E10) [20, 35, 41, 42] were relatively inefficient at capture of infectious virus and gp120 positive virions alike. This reflects early observations that 2F5 binds slowly to functional Env (several hours) and induces conformational change that ultimately leads to shedding of gp120 [43]. Furthermore, nnAbs 4B3, 3D6, 7B2 and F240 targeting gp41 C1 or non-trimeric forms of gp120 (b6) captured only a minority of infectious virus, indicative of the occlusion or absence of their cognate epitope on infectious virions and lack of co-expression with functional forms of Env.

The high correlation between capture of gp120 containing virions and capture of infectious virus by nAbs to gp120 may provide a correlate of the nature of infectious virions whereby the majority of gp120s are attached to functional particles. Therefore, although processing of Env can give rise to monomeric, dimeric and trimeric forms there appears to be limited incorporation into infectious virus. This was confirmed by native and SDS PAGE that indicate the predominant form of virion associated Env was trimeric gp120/gp41, concordant with previous studies [35]. These observations are dependent on the known specificity of individual mAbs and their ability to discriminate between functional and non-functional Env forms. We cannot exclude that particles predominantly expressing functional Env forms may still express non-functional forms at a level below our sensitivity of detection or at a frequency too low to facilitate effective viral capture.

Sequential viral sorting was applied to further interpret the distribution of Env structures within a purified HIV-1 population using mAbs targeting key structures (Table 2): b12 binding gp120; 2F5 specific for MPER; and 4B3 specific for gp41 C1. Pair-wise sorting experiments using these antibodies revealed three distinct but overlapping subpopulations (Fig. 8a). These data suggest discrete partitioning of Env forms across the viral population: virions homologous for functional Env, those heterologous for functional and non-functional Env and those homologous for non-functional forms. It is unclear whether the different forms represent differences in viral assembly/maturation, transitional states of Env degradation, or aspects of both. Previous studies indicated that released virus has equivalent molar amounts of integrated gp41 and gp120 that are relatively stable [44], however the half-life of infectious virions at 37 °C is estimated to be in the order of 6–18 h [45]. Virus used in most of these studies was harvested 72 h after media change; nevertheless, the timing of virus harvest (24 vs 72 h) did not significantly affect the proportions of virus captured. It is interesting to note that virus devoid of gp120 can be dual positive for MPER and gp41 C1 or single positive for gp41 C1. 2F5 is thought to preferentially bind gp41 monomers [20], while 4B3 is likely similar to other C1 gp41 antibodies that bind all multimerizations of gp41. Therefore 2F5/4B3 positive stumps likely encompass gp41 monomers as well as multimeric forms, while 4B3 single positive stumps reflect only multimeric forms.

The close Gag-Env association during particle release is thought to constrain Env subunits into a more widely distributed conformation where gp41 epitopes are more exposed; while these epitopes become masked during particle maturation [46, 47] and/or cleavage of gp160 [41, 42]. Thus virions triple positive for gp120 (b12), MPER (2F5) and gp41 C1 (4B3) (16.6 %) may represent a fraction of immature particles and/or those with uncleaved gp160 within the total population, which all mAbs were able to deplete. Alternatively this triple positive population may represent an intermediate state of envelope subunit dissociation before total loss of gp120 [13, 45]. Future experiments are needed to distinguish the relative contributions of these two options to this heterologous subpopulation.

To further interpret the distribution of gp120 structures within a homogenous viral population sequential sorting was performed using mAbs targeting functional (b12) and non-functional gp120 forms (b6); b6 mAb is thought to preferentially recognize non-trimeric gp120 or immature trimers. Given that b6 single positive virus would be non-infectious, reflecting virus displaying only non-functional Env, then of the total infectious b12 positive virions, 64 % would be single positive for b12 and 36 % dual positive (b6+ b12+). These data are very similar to our observation that b6 captures 32 % of infectious virus while b12 captures >87 % (Fig. 6). This provides a possible explanation as to why b6 fails to block b12 neutralization despite targeting broadly overlapping epitopes in the CD4bs of gp120 [15].

Our data concord with previous reports that defective Env structures are thought to make up a large proportion of the total virions [13, 48]. Removal of the non-infectious virions that express the 4B3 or 3D6 epitopes depleted only a small fraction of virions susceptible to neutralization by 2G12, b12, or 1F7. This confirms that virions expressing gp120 epitopes vulnerable to neutralization are largely distinct from non-infectious virus expressing accessible gp41 C1, most likely representing Env ‘stumps’which consist only of gp41 without any associated gp120 [13]. These data complement the observations that 4B3 bound <8 % infectious virions. The impact of 4B3 and 3D6 depletion on the neutralization activity of 4E10 and 2F5 likely reflects the non-infectious population that expresses both gp41 C1 and MPER epitopes (Fig. 8) acting as a potential sink for MPER targeting antibodies and reducing the fraction free to neutralize by binding Env fusogenic intermediates following CD4 interaction on target cells. However the relatively limited impact on activity suggests this is unlikely to play a substantial role in vivo.

Viral capture of infectious, functional virions by nAbs concords with the accessibility of these sites revealed by recent structural data on soluble stabilized Env trimers [49–51]. Although some differences are expected due to variation in clade or effect of stabilizing mutations present in native-like soluble trimers, we find only minor variations, particularly in relation to b12 capture [52]. However these are in accord with the recently described and more closely matched stabilized B41 soluble clade B trimer [53]. The previously described occlusion of non-neutralizing epitopes on virion expressed functional trimers [48, 54] is consistent with the inefficient capture of infectious virions by nnAbs observed in this study. Recent observations that vaccination with virus-like particles, depleted of non-functional Env by enzymatic digestion, can elicit Tier 2 neutralizing responses confirm the importance of nAb recognition of native functional Env [55].

Our observation that a significant fraction of infectious virions were exclusively captured by nAbs is indicative of a homologous distribution of functional trimeric Env forms. Indeed, heterologous distribution of functional and non-functional forms represented a minor fraction of the total viral population, the majority of virions being homologous either for functional or non-functional forms of Env. This may have important implications for the design of effective antibody based prophylactic vaccines targeting cell free virions. The observation that a significant proportion of virus displaying functional Env was negative for binding of nnAbs brings into question potential mechanisms of direct functional interaction of such antibodies with free infectious virions. Proposed mechanisms of inhibition by nnAbs include: antibody dependent viral capture; aggregation; mucus retardation; transcytosis inhibition; complement mediated cytolysis; and cell mediated phagocytosis (ADCP) [56]. All are predicated on their binding to infectious virions. While the smaller population of infectious virions heterologous with respect to functional and non-functional forms of Env would be susceptible to these mechanisms, the majority of virions homologous for functional Env would be unimpeded by such response.

Should this be the case in vivo then protective vaccines targeted against cell free virions may critically depend on recognition of functional trimers. This does not however exclude a potential contributing role for nnAb in elimination of infected target cells through ADCC or ADCP. Indeed most protective nnAbs described to date react with viral epitopes expressed on the surface of infected cells rather than the surface of virions [57].

## Conclusions

In summary, the key finding of this work is the recognition that the majority of infectious virions within a viral population are homologous with respect to expression of functional Env, with a smaller population heterologous for functional and non-functional forms. These observations argue against a random distribution of non-functional forms of Env across an infectious viral population. The predominant homologous distribution of functional Env predicates that recognition of functional Env is essential for antibody binding to the majority of infectious virions, a trait thought to be synonymous with neutralization. These observations should be taken into account in the design and selection of prophylactic vaccines targeting infectious virions.

## Methods

### Cells and reagents

TZM-bl and PM-1 cells were obtained from the NIH-AIDS Research and Reference Reagent Program. Antibodies 2F5, 2G12, 4E10, PG9, PG16, and 17b were obtained through the Center for HIV and AIDS Vaccine and Immunology (CHAVI). Antibodies 5F3, 4B3, 3D6, and 1F7 were obtained from Polymun Scientific, GmbH (Austria). Antibodies b12, b6, VRC01, and VRC03 were obtained from Dennis Burton, Scripps Research Institute (La Jolla, United States). The following reagents were obtained through the NIH AIDS Research and Reference Reagent Program, Division of AIDS, NIAID, NIH: Soluble Human CD4 from Progenics, TZM-bl from Dr. John C. Kappes, Dr. Xiaoyun Wu and Tranzyme Inc. T cell line PM-1 was donated by Dr. Marvin Reitz. HIV-1_BaL_ was donated by Dr. Suzanne Gartner, Dr. Mikulas Popovic and Dr. Robert Gallo. Non-specific human IgG antibody was obtained from Sigma. Monoclonal antibodies against human CD45 and HLA-DR were obtained from Santa Cruz Biotechnology.

### Viral culture and purification

HIV-1_BaL_ and HIV-1_Bx08_ viral stocks were produced in PM-1 T-cells to reduce potential influence of donor variability when using PBMC grown virus. Briefly, 2 × 10^6^ PM-1 cells were re-suspended in 1 ml of fresh medium (RPMI + 10 % FCS + 2 mM l-glutamine + 100 U ml^−1^ Penicillin + 100 μg ml^−1^ Streptomycin) and 1 ml of infectious HIV-1_BaL_ stock was added. After 3 days 2 × 10^7^ fresh, uninfected PM-1 cells were added to the culture in 20 ml of fresh medium. After 7 days, 1 × 10^8^ additional PM-1 cells were added to the culture and the total volume made up to 150 ml with fresh medium. The culture was further expanded on day 10 by the addition of 150 ml of fresh medium, taking the total volume to 300 ml, and was harvested on day 13 by centrifugation at 2500 rpm for 15 min. Virus was inactivated with a final concentration of 1 mM aldrithiol (AT-2, Sigma, UK) for 2.5 h at 37 °C and frozen at −80 °C. Virus concentration of this stock was greater than 767 ng ml^−1^ p24 measured by HIV-1 p24-gag ELISA (Frederick) according to the manufacturer’s instructions, and TCID_50_ ml^−1^ on PM-1 cells was 5.2 × 10^6^. Stocks of the transmitted/founder (T/F) clade C virus CH162 (accession number KC156126, kindly provided by Dr. Christina Ochsenbauer) were produced by transfection of the proviral plasmid into 293FT cells with PEI:DNA ratio of 4:1, for 4 h. After 48 h, supernatant was removed and transfected 293FT cells were co-cultured with C8166-R5 T cells. After a further 4 days, C8166-R5 cells were centrifuged at 300×*g* and co-cultured with uninfected C8166-R5 cells for 72 h. Virus was then harvested as described above.

Microvesicle depleted HIV-1 virion stocks were purified as previously described [39, 58] with some modifications. Briefly, viral stocks were concentrated over a 17–25 % sucrose in PBS cushion in a Beckman SW55Ti rotor. Virions were resuspended in PBS, supplemented with 1 mM EDTA and 0.1 % BSA (binding buffer) with CD45 labeled magnetic beads (Miltenyi Biotec, Germany) at 4 °C with gentle mixing for at least 4 h. Antibody conjugated beads were used at a concentration of 1 μl of beads per 50 ng of p24 in the viral stock. Depletion of CD45+ve microvesicles was performed over LD columns. Microvesicle depleted viral preparations were collected as the flow through from the column and re-concentrated over 25 % sucrose cushions in a Beckman SW55Ti rotor and resuspended in PBS.

### Immuno-bead viral capture assay (IB-VCA)

Purified virions, used at a concentration of 500 ng ml^−1^ p24, were combined with the antibody of interest at a concentration of 10 μg ml^−1^ and brought to a final volume of 75 μl binding buffer then incubated at 37 °C for 1 h. 25 μl of protein G conjugated magnetic beads (Miltenyi Biotec) were then added to the antibody coated virions and incubated at 4 °C for 30 min. Separation of labeled fraction was performed on magnetic μ-columns. Fresh columns were primed with 1 ml binding buffer, and then the viral preparation was applied. Three washes of 100 μl of binding buffer removed all unlabeled virions. The eluted portion was collected as the negative fraction. The column was then removed from the magnet and washed four times with binding buffer. This portion of eluent was collected as the positive fraction. Virions were then lysed with Triton-X100 at a 1 % concentration with freezing. Each separation was performed in triplicate and at least three experiments were performed for each condition.

The percentage of binding was determined by ELISA where the amount of either p24 or oligomeric Env present in the positive fraction was expressed proportionally to the total of the positive and negative fractions. ELISA for Env content of BaL was performed by coating 96-well plates with 5 μg ml^−1^ of *galanthus nivalis* lectin (GNA) (Sigma-Aldrich), followed by the lysed virion preparations [59]. Detection was done with 5F3 monoclonal followed by HRP conjugated mouse anti-human IgG. Colorimetric signal was produced using the TMB substrate (Pierce) and absorbance read at 450 nm using a microplate reader (BMG Labtech). The 5F3 monoclonal recognizes a linear gp41 epitope adjacent to the N-terminal fusion peptide, and preferentially binds trimeric and dimeric forms of Env [60], as GNA does not bind free gp41, this ELISA effectively detects oligomeric forms of Env. A more sensitive ELISA was used for quantification of CH162.c Env. 96-well plates were coated with sheep polyclonal capture antibody D7324 (Aalto Bio Reagents). Sorted viral preparations were then immobilized and detected with VRC01 followed by a secondary amplification with biotinylated goat anti-human IgG followed by Streptavidin-Poly-HRP40 and developed with TMB. The ELISA for p24-gag was performed using prepared quantification kits from the AIDS Vaccine Program, SAIC, Frederick, MD carried out according to the manufacturer’s instructions.

### Immuno-bead infectivity depletion assay

Untreated viral stocks were titrated on TZM-bl cells and luciferase production was measured as described [38] to determine the 50 % tissue culture infectious dose (TCID_50_). 10^3^ TCID_50_ virions were incubated with the depletion antibody (10 μg) for 1 h at 37 °C, followed by addition of 50 μl protein G beads and 30 min incubation at 4 °C then depletion over μ-columns. Depleted virions were applied to TZM-bl cells in a twofold dilution series and luciferase production was measured after 24 h. Percentage depletion of infectivity was determined as the percent change in the area under the infectivity curve relative to the IgG isotype control depleted preparation. Control experiments were performed to demonstrate that no antibody was present in the eluted fraction.

### Native and SDS-PAGE and western blot

Native PAGE was performed as described previously with minor modifications [13, 48]. Briefly, inactivated virus was lysed for 10 min on ice with an equal volume of 2× lysis buffer (0.15 % Triton-X 100 in 1 mM EDTA and 1.5 M aminocaproic acid) and protease inhibitor cocktail (Roche Diagnostics, UK). Prior to loading on a 4–12 % Tris NuPAGE gel (Invitrogen) 2× sample buffer composed of 100 mM MOPS, 100 mM Tris–HCl pH 7.7, 40 % glycerol and 0.1 % Coomassie blue was added. Samples were separated on ice for 3 h at 100 V with cathode buffer containing 50 mM MOPS/50 mM Tris base pH 7.7 + 0.002 % Coomassie blue and same buffer without Coomassie as anode buffer. Proteins were transferred onto a PVDF membrane under wet conditions using transfer buffer containing 10 % methanol for 2 h at 20 V on ice. Excess Coomassie blue was removed by washing 3× with 100 % methanol for 10 min. Membranes were blocked in blocking buffer (4 % nonfat milk in PBS) for 1 h at RT and probed over night at 4 °C with cocktails of mAbs to gp120 (b12, 2G12, b6) or gp41 (2F5, 4B3, 3D6) diluted in blocking buffer. Membranes were washed 4× for 10 min with wash buffer (PBS + 0.05 % Tween) and incubated for 1 h at RT with goat anti-human Fc alkaline phosphatase conjugated secondary antibody (Sigma, UK) diluted 1:2000 in blocking buffer. The membranes were washed and developed with Sigmafast BCIP/NBT (Sigma, UK) for 5–10 min at RT. Band intensities were quantified using FIJI/ImageJ v1.48 g gel analysis tools. Samples were prepared for SDS PAGE analysis by concentration of antibody depleted virus fractions using 100,000MWCO vivaspin 500 microcolumns. Total protein concentration was measured by absorbance at 280 nm on a Nanodrop Spectrophotometer. 25 μg total protein was loaded for each sample.

Concentrated virus samples are incubated in NuPAGE Reducing Agent and Tris–Glycine Sample Buffer (2×), at 95 **°**C, for 2 min, prior to loading on 4–12 % Tris–Glycine gels (Novex). Protein samples were separated at room temperature using Tris–Glycine SDS running buffer (Novex) at 150 V for 90 min, transferred to PVDF membrane using semi-dry transfer in 39 mM glycine, 48 mM Tris, with 20 % methanol. For detection of gp120 HIV Env species, membranes were blocked in 3 % BSA in TBS and 0.1 % Tween for 1 h at room temperature with constant agitation. Env proteins were detected with b13 hybridoma supernatant (a kind gift from Prof. George Lewis) overnight at 4 °C, followed by anti-mouse biotin at 1:5000 dilution (Southern Biotech) and streptavidin conjugated to Alkaline-phosphatase at 1:1000 dilution (MabTech), each for 1 h at room temperature with constant agitation. Membranes were then developed with BCIP/NBT (Sigma, UK) for 30 min at room temperature. gp41 proteins were detected by blocking membranes with 1 % BSA in TBS and 0.1 % Tween and detected with a cocktail of 2F5, 4E10 and 5F3 (1 μg ml^−1^), followed by anti-human biotin at 1:1000 dilution (MabTech), Streptavidin conjugated to Alkaline-phosphatase and BCIP/NBT as described above.

### Visualization of virion depletion by nanoparticle tracking

The number of virus particles present in control (IgG) and antibody depleted virus samples was determined by nanoparticle tracking analysis using a Nanosight LM10 instrument as previously described [34]. Samples were diluted to obtain particles counts between 2 × 10^8^ and 9 × 10^8^ particles/ml to ensure accurate counting. For each sample, particle images were captured for 60 s in triplicate. Particle counts were assessed using screen gain of 10, and detection threshold of 4.0.

### Viral neutralization assay

Depleted HIV-1_BaL_ virions were prepared as for the infectivity depletion assay with either 4B3 or 3D6. Serial dilutions of antibody were pre-incubated with untreated or depleted virions at a range from 20 μg ml^−1^ to 156 ng ml^−1^ of nAbs of interest for 1 h at 37 °C. Virus-antibody preparations were then applied to TZM-bl cells and incubated for 24 h at 37 °C after which cells were lysed and the Luciferase activity was measured as relative light units by a luminometer. 50 % inhibitory dose (IC_50_) was determined as the concentration of antibody which luminescence was reduced by 50 % compared to virus control wells.

### Statistical analysis

Statistical comparisons were made using GraphPad Prism v6.00. Wilcoxon signed rank test was used to determine significance of differences in viral capture assays. All correlations were determined by two-tailed Spearman correlation analysis. 1-way ANOVA was used to determine the significance of effects of harvest time on viral capture and the variance in IC_50_ in neutralization assays after antibody depletion.

## Additional files

**Additional file 1: Figure S1.** Depletion of infectious particles with a panel of Env-specific mAbs. Infectious stock of HIV-1_BaL_ was treated with the specified antibody, and infectivity depleted as described in Figure 6 legend. Data represent the mean of triplicate experiments.
**Additional file 2: Figure S2.** Depletion of infectious particles from primary HIV-1 isolates. (A) Depletion of HIV-1 strain Bx08 infectious particles with a panel of Env-specific mAbs. Infectivity depletion was performed as described in Figure 6 legend. Data represent the mean of duplicate experiments. HIV-1 strain CH162.c was analyzed for correlation between (B)  % of p24 capture and  % infectivity depletion, (C)  % of p24 and  % of gp120 capture, and (D)  % gp120 capture and  % infectivity depletion. Spearman correlation coefficients and associated p values are shown. Error bars indicate the standard deviation of 3 or 4 independent experiments.
**Additional file 3: Figure S3.** Monoclonal antibodies are completely retained by the magnetic column. Monoclonal antibody b12 was incubated with Protein G magnetic beads in the absence of virus, and passed through a magnetic column. The resulting flow-through was added to HIV-1_BaL_ and titrated on TZM-bl cells to determine if any residual inhibitory activity had passed through the column. Luciferase production was measured after 24 h as a function of viral input.

## Abbreviations

bnAb: broadly neutralizing antibody
CD4bs: CD4 binding site
CD4i: CD4-induced
C1: cluster I
Env: envelope glycoprotein
VCA: immuno-bead virus capture assay
IC_50_: inhibitory concentration 50 %
MPER: membrane proximal external region
mAb: monoclonal antibody
nAb: neutralizing antibody
nnAb: non-neutralizing antibody
