# Supplementary figures and images for: Discrete partitioning of HIV-1 Env forms revealed by viral capture
Source: Retrovirology. 2015 Sep 24;12:81. doi: 10.1186/s12977-015-0207-z (PMC4581120; doi:10.1186/s12977-015-0207-z)

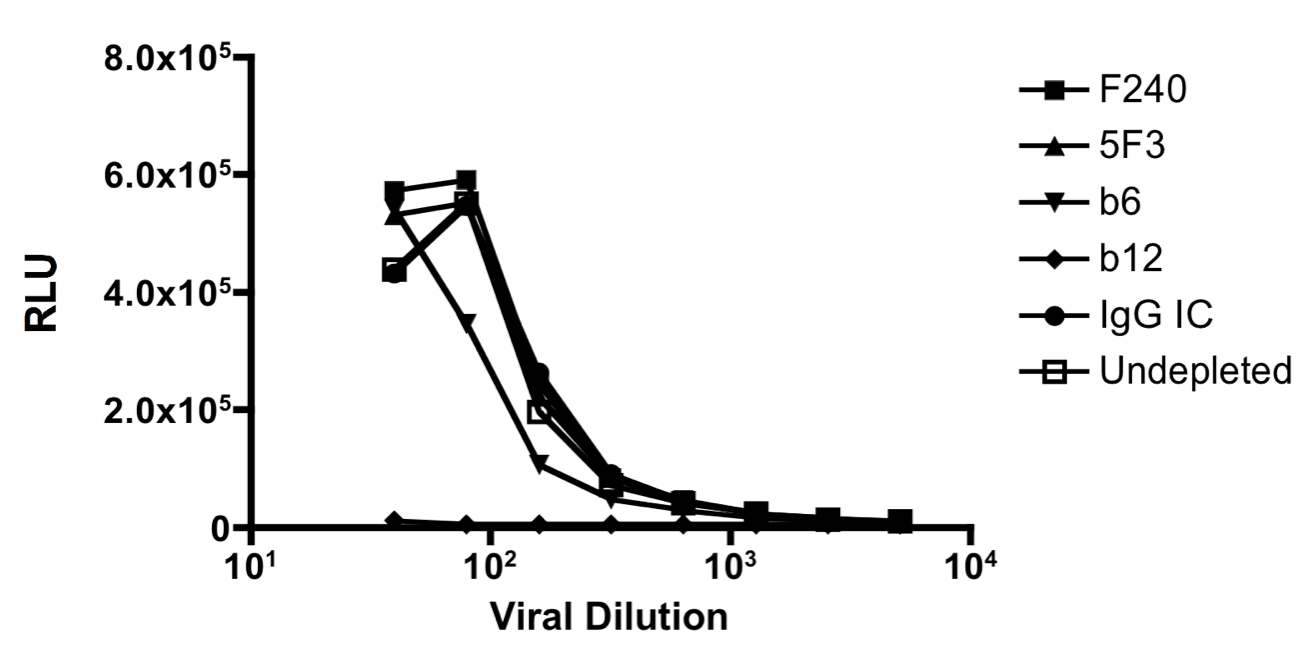

Supplement: Supplementary file 1 — Additional file 1: Figure S1. Depletion of infectious particles with a panel of Env-specific mAbs. Infectious stock of HIV-1BaL was treated with the specified antibody, and infectivity depleted as described in Figure 6 legend. Data represent the mean of triplicate experiments. [file 12977_2015_207_MOESM1_ESM.tif]

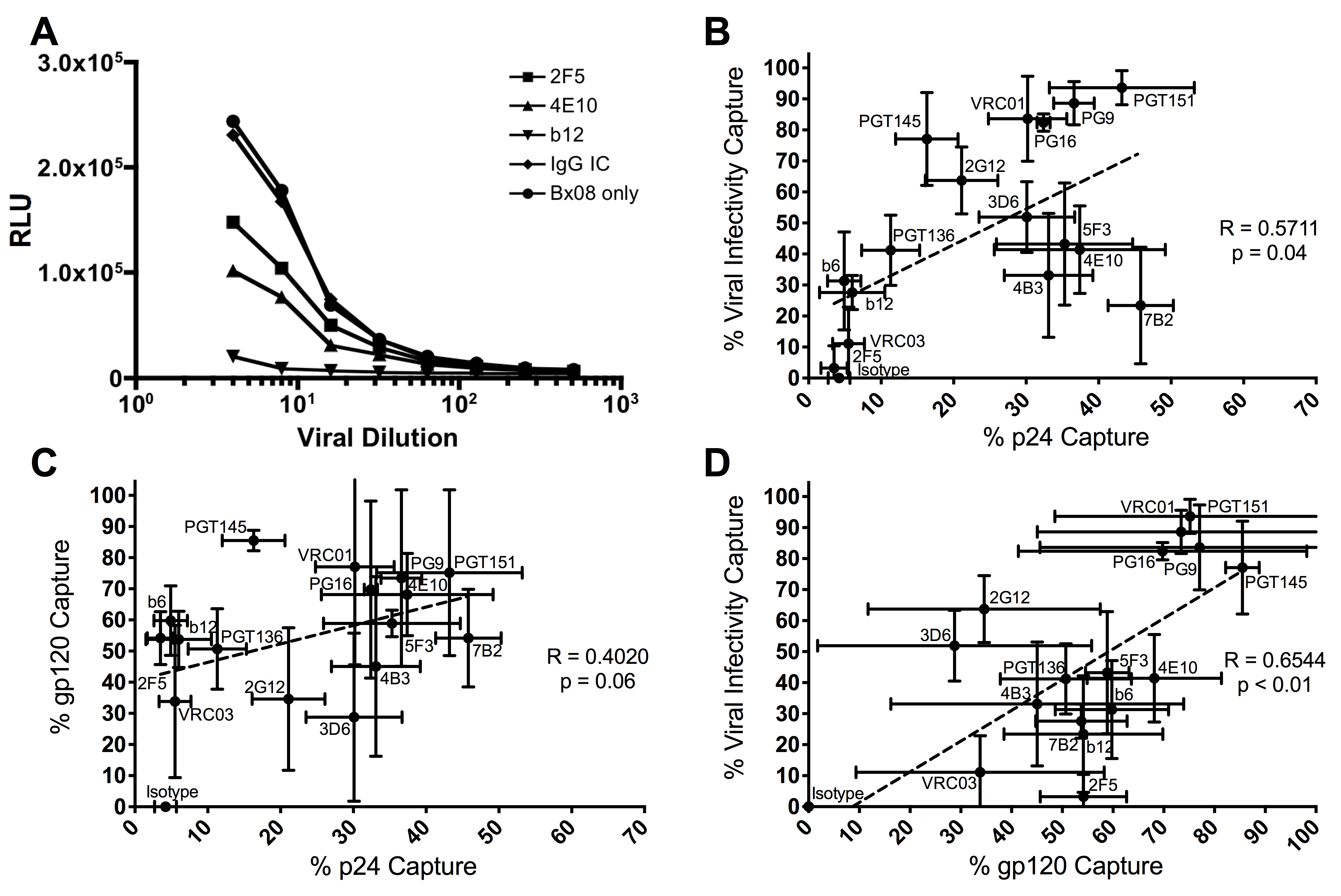

Supplement: Supplementary file 2 — Additional file 2: Figure S2. Depletion of infectious particles from primary HIV-1 isolates. (A) Depletion of HIV-1 strain Bx08 infectious particles with a panel of Env-specific mAbs. Infectivity depletion was performed as described in Figure 6 legend. Data represent the mean of duplicate experiments. HIV-1 strain CH162.c was analyzed for correlation between (B) % of p24 capture and % infectivity depletion, (C) % of p24 and % of gp120 capture, and (D) % gp120 capture and % infectivity depletion. Spearman correlation coefficients and associated p values are shown. Error bars indicate the standard deviation of 3 or 4 independent experiments. [file 12977_2015_207_MOESM2_ESM.tiff]

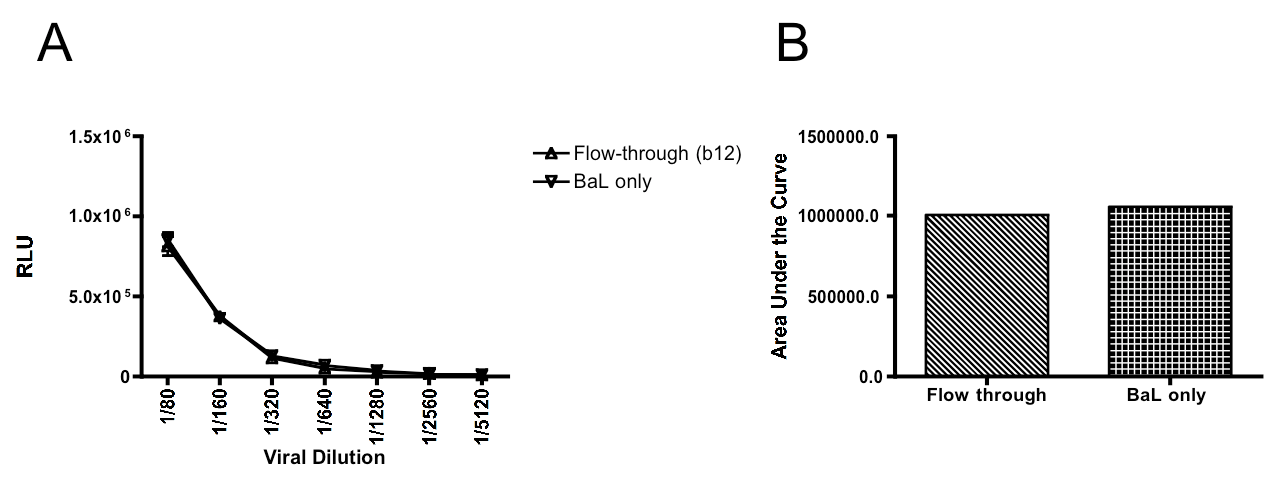

Supplement: Supplementary file 3 — Additional file 3: Figure S3. Monoclonal antibodies are completely retained by the magnetic column. Monoclonal antibody b12 was incubated with Protein G magnetic beads in the absence of virus, and passed through a magnetic column. The resulting flow-through was added to HIV-1BaL and titrated on TZM-bl cells to determine if any residual inhibitory activity had passed through the column. Luciferase production was measured after 24 h as a function of viral input. [file 12977_2015_207_MOESM3_ESM.tif]
